# Appendicitis, Etc., Complicating Pregnancy, Labor and the Puerperium

**Published:** 1902-01

**Authors:** Samuel F. Brothers

**Affiliations:** New York City, Visiting and attending gynecologist to the Metropolitan Hospital and Dispensary, New York.


### Appendicitis, Etc., Complicating Pregnancy, Labor and the Puerperium.

By SAMUEL F. BROTHERS, Ph. G., M. D., New York City,
Visiting and attending gynecologist to the Metropolitan Hospital and Dispensary, New York.

UP TO the beginning of 1897 only fifteen cases of appendicitis complicating the reproductive state, were said to have been recorded. Pinard, in March, 1898, collected 45

1. Read before the New York County Medico-Pharmaceutical League, September 26, 1901.

cases, these probably including all the recorded cases in Europe as well as this country. Until a very recent date, nearly all these cases were included in the terms cecitis, pericecitis, typhlitis, pelvic abscess, local peritonitis and gangrene of the cecum.

The case now recited was brought to my attention on April 30, 1900, when I was requested to call on Mrs. B. M., in her fourth pregnancy. She was at this time in the latter part of the sixth month. I found her with a temperature of 101° F., with the usual pain and tenderness in the right iliac fossa. She said that she "had the same pain on the right side in her last pregnancy," lasting until a month after the birth of the child. On examination, the anterior cervical and uterine wall was found soft and knobby, this feeling extending outward toward the right fossa. The patient was perfectly self-possessed. The focal intensity of pain externally, corresponded to the base of the appendix, between the anterior spine and the umbilicus; the appendix could almost be felt. There was distinct circumscribed muscle tension. On the third day an iliac tumor was appreciable. On the fifth day dulness could be detected, but no fluctuation as yet. There was moderate vomiting.

Typhilitis was excluded, as the swelling was not '"boggy" and temperature and pain were marked. Appendicial, renal and biliary colics were excluded by the febrile condition present and the absence of excruciating pain. As there was no history of spinal trouble, psoas abscess was not considered. Tubal and ovarian disease were excluded by the absence of any history of menstrual complaints and the want of indications during bimanual and rectal palpation. The intestinal obstruction was not marked. Typhoid was excluded by the absence of decided prostration, and of furred tongue, headache and excessive frequency of pulse. The intermittent, intense pain of iliac neuralgia was also absent.

The diagnosis of appendicitis having been therefore settled, narcotics were ordered until the next day, the 31st, when the temperature was found reduced to 99 1-5°; pulse, 95; respiration, 23. Twenty-four hours later, May 1, intermittent pains began; the fetal movements on the right side caused pain; temperature, 99°. On the next day, May 3, a vaginal discharge of pus occurred, having a disagreeable odor, but its site could not be located; temperature, 98½°. This discharge had been noticed since the preceding three days; the opiates were continued in moderate doses. She said that her last child was born thirteen months ago, and her trouble on the right side had continued from the third month of that pregnancy, until two months after the birth of the child.

Two days later, May 5, I was again hurriedly called to find, on examination, that prémature labor had set in; her pulse registered 130 beats to the minute; she had had steady intermittent pains for the last four hours. The os was found dilated to the dimensions of a silver dollar; the head was presenting, with the right foot alongside of it. The bag of waters burst at 11 p.m., the position being now left occipito-anterior. Birth occurred soon after, but the infant was almost lifeless, though breathing quietly. It expired ten hours later.

Placental removal was tedious, but after laborious twisting of the adherent membranes, apparently complete. No pain now, but temperature, 103°; pulse, 135; quinine sulphate prescribed. By next day, May 6, her temperature had become subnormal, $97\frac{1}{2}$° F., but pulse was 100. On the 7th, a day later, headache was complained of, the temperature was normal, yet pulse continued the same. In another twenty-four hours fever again developed, 102°; the uterine discharge was suppressed, and a disagreeable odor developed; a piece of membrane had been passed during the day.

Curettage was performed, and large, frequently repeated vaginal douches were ordered. The uterine body was extremely anteflexed upon the cervix. Another day, and her temperature and pulse had returned to the normal. Within the next four days it rose and then fell again to the normal, after which recovery was considered complete. After a full week had elapsed, however, I was again called, and found that although the patient had been about already, there was found on examination a distinctly circumscribed mass in the *left* pelvic fossa, with hectic symptoms. This was diagnosticated pelvic cellulitis. This developed into a fluctuating abscess within three days, pointing toward the abdomen; when operated, a large amount of pus was discharged, after which recovery progressed uninterruptedly.

I have attended two other cases, in one of which Dr. E. A. Gallant was called in.

In the Transactions of the Mississippi Valley Medical Association for 1899, Dr. Lewis Schooler, a surgeon, says in an article entitled, What becomes of the medicinally treated cases of appendicitis:

That some die from obstruction of the bowels caused by perforation of the appendix, and others from heart failure, peritonitis or sepsis; that a few get well by permanent disappearance of the symptoms; and that others are spontaneously cured by rupture into the bowel, vagina, bladder or externally.

Dr. J. W. Thomason, of Huntsville, Texas, in the second volume of the *Medical Record* for 1895, page 407, says:

Many cases of appendicitis will recover by natural processes and may be influenced favorably by medical treatment only. My own professional experience, he said, has convinced me of this fact, and that such is a common experience is one reason why surgical treatment is so often postponed longer than it should be. Then, too, delay in these cases does not always prove fatal. Such a case reported by Dr. Mundé was of a woman, eight months pregnant, the appendicitis being followed a week later by the delivery of a dead fetus. The abscess was diagnosticated a day after this, the operation performed six days later, and yet a good recovery resulted. On the other hand, operations reported, seem not to have been followed by such results as would justify surgical measures without hesitation.

Dr. L. L. McArthur, of Chicago, reports two cases in the February, 1895, number of the *American Journal of Obstetrics:*

One was operated on the third day, was followed by a 4½ months abortion a day later, and death the following day. The other operated after the third week; a five months miscarriage two days later, and death on the fifth day. To illustrate the difficulty of diagnosis, Dr. McArthur reports a case of typical symptoms and a temperature of 105°, when several surgeons concurred in the diagnosis; an exploratory incision showed the appendix and other organs normal. A macerated fetus was aborted next day, and she recovered; this was considered the cause, as there was no appendicitis.

In the discussion which followed at the Chicago Gynecological Society, it was brought out that the troublesome feature in dealing with these cases was the fact that the pregnant womb generally forms one wall of the appendicial abscess. The irritation produces abortion and the contracting womb tears adhesions that walled off the abscess, permitting septic matter to penetrate the general peritoneal cavity. On this account one member suggested the induction of abortion first.

Another thought that in those cases where the womb forms one wall of the abscess, the induction of abortion would tear the adhesions also.

Dr. Thomason's conclusions were that it is best to be conservative.

In the same journal of October 26, Dr. P. F. Mundé says:

Dr. Thomason quotes the delay in operating in the case reported by me in the number of December, 1894, as part justification for his delay in operating the case related by him, where the patient recovered without operation, and was delivered at term of a healthy child. While this result undoubtedly warranted the delay, and eventually the non-performance of an operation, I would not be understood as advising such delay in operating in other similar cases. In the case I saw with Dr. Hochheimer, he delayed the operation only four days, and I stated, I think, that in a future similar case I would open the abscess at once, as soon as a reasonable probability of its existence could be settled without reference to the pregnancy or the impending or completed delivery. I would rather take the chances of puerperal infection from the abscess than of its unexpected rupture at any moment into the peritoneal cavity. Would that the question of diagnosis were as easily settled.

Dr. Thomason quotes several authors who report difficulty in diagnosis and unfavorable results from early operations, the women aborting and dying several days later. They do not say, however, nor can they say with any certainty, how long the acute inflammation of the appendix (probably with perforation already accomplished) had existed before the patient was seen by the operator.

In the case I relate, he continues, Dr. David Franklin asked me to see with him Mrs. ——, a multipara, who had been confined by a midwife two days before of a dead anencephalic fetus at term. The placenta failed to come away, but, in the doctor's absence, Dr. Chas. M. Ford was called, and detached it from the right side, and delivered it. Within twelve hours she began to vomit, the abdomen became tympanitic and right iliac pain developed.

When I saw her, pulse was 63, strong, but dicrotic; temperature and respiration normal; no particular sign of collapse; no perceptible dulness or resistance over the appendix; vaginal examination proved nothing. The patient said that her health was good before the confinement, but admitted that during her last three or four pregnancies, up to the fourth or fifth month, she had more or less pain, which gradually wore away. Her husband added that sixteen years ago she was very ill with a similar pain.

I diagnosed, says Dr. Mundé, besides the recurrent appendicitis, a probable rupture of an adhesion, produced either by the uterine contractions or possibly during the detachment of the placenta. The diagnosis and former probability were imparted to the husband, the latter possibility to Drs. Franklin and Ford privately. A perforation of the appendix was not then entertained, since there was no sign of general peritonitis. The vomiting, however, I decided to be undoubtedly reflex, from a local peritonitis, and I advised first controlling the vomiting by hypodermics of morphine over the epigastrium; and reducing the tympanites by ox-gall, glycerine and turpentine enemata; so as to enable us to arrive at a more positive diagnosis as to the actual presence of an appendicial abscess, and the advisability of operative interference.

The patient at that time did not seem in a good condition for anesthesia and laparatomy. Nutrition by the rectum was also recommended. Three days later much improvement was reported, but the same morning tympanites returned, the temperature and pulse went up to 101° and 112 respectively.

That afternoon I found that the stomach was good, but the tongue coated; strength still good; no decided local dulness or resistance; lochia offensive. Decided efforts were now made to empty the intestines of gas and whatever feces they might contain by means of $\frac{1}{2}$ grain doses of calomel every half hour until twenty had been taken, and followed by dram doses of Rochelle salts and ox-gall enemata if necessary. Unless she improved by morning, an exploratory incision was suggested.

As the tympanites still persisted, and there had been no movement, I recommended, the next morning, puncturing the most distended coils of intestine with several hypodermic needles,—a proceeding repeatedly employed by me with benefit and no danger,—and arranged to operate later.

With the assistance of Drs. Franklin, E. Sternberger, Ford and Richards, I made the usual right lateral incision and soon discovered from the infiltration of the tissues, that there was deep-seated inflammation. On incising the peritoneum a large quantity of gas, and solid and fluid feces escaped, showing that a perforation of the appendix had taken place.

The general peritoneal cavity was apparently entirely shut off by the adherent intestines. Turning the patient on the right side, I gently irrigated the abscess cavity with warm sterilised water, and packed it loosely with iodoform gauze. The patient rallied from the operation remarkably well, and during the next twenty-four hours enormous quantities of gas escaped and fluid feces oozed through the dressing and were passed per anum. The temperature was normal on the next day, and the patient's condition fairly good, except an unpleasantly rapid, although good, pulse. The deep dressing was not disturbed for fear of rupturing fresh adhesions. Stimulants *ad libitum* were ordered, chiefly champagne. After a good night, however, a change for the worse took place. The pulse went up to 140 and became thready, and after noon she seemed almost moribund. The dressing was changed, oxygen inhalations were ordered, stimulation hypodermatically, and the like. Three days later she died from exhaustion.

In consequence of the recurrent attacks, undoubtedly the appendix was adherent, and in a soft, friable condition at the time of the last labor; and during or immediately following the labor, perforation took place, and fecal matter was discharged into a cavity sealed off by the previous attacks. This perforation was the cause of the reflex vomiting, and the intestinal paresis causing tympanites. The diagnosis of appendicitis was made twenty hours after perforation had occurred, when I first saw her. The diagnosis of perforation, is, after all, the chief difficulty.

To prove the possibility of spontaneous recovery after perforation, Dr. Mundé related the case of a male in whom the appendix broke away piecemeal, on attempting to tie it off, its fragile character, showing that it had been perforated for some time previously.

He concludes:

The disease should be treated entirely regardless of the existence of pregnancy. I do not agree with those gentlemen, therefore, who would induce abortion or premature labor during, and on account of, appendicitis.

Dr. S. Marx, in the same journal of June 18, 1898, in a clinical report, says that "there was reason to think that the congestion of the system and the vicious condition of the blood associated with pregnancy acted as a predisposing cause of appendicitis." He thought that the diagnosis was "not of much importance, because if the symptoms were sufficiently severe the treatment was operative, whether the appendix or the uterine appendages were the seat of the inflammation."

On the other hand, operations reported, seem not to have been followed by such results as would justify surgical measures without hesitation. In one case Dr. Willy Meyer removed an eight-inch appendix where the disease developed six hours after a normal labor.

Another case was a four months primipara, operated by Dr. Gerster. Recovery was satisfactory, but, although the delivery was normal, forceps was used with the idea that it would take the strain of the second stage off of the abdominal scar. A few hours later she experienced much pain about this scar, apparently from the rupture of an abdominal adhesion band.

The third case was a di-para two-and-a-half months after a suppurating ovarian dermoid had been removed. The appendicial symptoms developed after labor. She recovered, refusing operation, but subsequently allowed it, during a recurrence. The appendix was firmly imbedded in the posterior wall of the suppurating mass; recovery ensued.

The fourth patient was a primipara in the maternity hospital, who had an attack of peritonitis three years before; at the seventh month of pregnancy she began to complain of pain over the appendix, but the pulse and temperature were normal. The treatment consisted in catharsis, the use of the ice-bag and rest in bed. At the operation, the right tube and ovary constituted part of the abscess-sac and the appendix was necrotic. Twenty-four hours later labor set in, and a living child was delivered. The temperature and pulse rose rapidly, general septicemia was apprehended and she was given 10 c.c. of Gibier's antistreptococcus serum, with the happiest result. The appendix came away on the fifth day; on the twelfth she began to have chills and fever, and to develop a sero-purulent peritonitis. After an extensive operation convalescence was slowly established. Meanwhile the serum was continued in small doses.

Dr. Marx said that where the uterus formed part of the abscess-wall, he delivered the woman at the beginning of labor by manual dilatation of the cervix, version or forceps delivery, and immediately tamponned the utero-vaginal tract.

In the fifth and last case recorded, the abscess cavity was encapsulated. The parts were thoroughly washed with hydrozone and a gauze drain was inserted. All of the symptoms improved; on the sixth day it was decided to suture the wound. She suddenly developed symptoms of intestinal obstruction, and exploration under ether revealed an obstruction in the ascending colon, due to a constricting band. Pus was found coming from the general peritoneal cavity, so multiple incisions were made, a gauze drain was inserted and doses of Marmorek's streptococcus serum administered. A fatal termination occurred in forty-eight hours. In his puerperal streptococcus cases the serum had invariably failed; when it acted well, the effect was observed quickly; large doses of the serum produced headache, nausea and vomiting, with rise of temperature.

Dr. Boldt said, during the discussion, that he had once a case complicating extrauterine pregnancy, the diagnosis being made during the operation for the ectopic gestation.

Dr. Wells did not think a recent abdominal scar should indicate instrumental delivery. He had delivered from six weeks to four months after abdominal section without any injury to the scar.

Dr. Howard Crutcher, of Chicago, (*Medical Record*, September 26, 1896,) relates the case of a seventeen-year old, unmarried female patient of Dr. Frank H. Waters, who was curetted after a two-months' abortion; after general sepsis developed, operation showed a perforated gangrenous appendix, adherent to the right tube and uterine fundus. A fatal result ensued.

Up to December 5, 1896, (same journal) Dr. Robert Abrahams stated before the New York County Medical Association, that only 11 cases had been placed on record; 10 suppurative, 1 catarrhal. He added 4 new ones, in which both mothers and infants lived. In his first case, the 7½ months infant lived six days, the mother recovering spontaneously. The second case suffered from two recurrent attacks since the child-birth, but refused operation. The other two also recovered without operation. He thought that obstinate constipation might be a cause, as it was present in all but one of his cases. The pathology only differed from the same condition without pregnancy in that the enlarging uterus would affect adhesions. In differential diagnosis, a tubal pregnancy only lasted four months before rupture. As to hematocele, in only one of the cases on record had a tumor of doughy feel at the vault of the vagina, proven to be appendicitis. Inflammation of the pelvic organs, ovaritis and salpingitis, was almost always infectious in origin. Floating kidney and renal calculus might occasionally be mistaken for it.

As to prognosis, there had been seven deaths out of ten suppurative cases. All cases of catarrhal appendicitis had recovered. Of operated women, only one child had lived; the rest died before or after operation on the mother. Of the whole fifteen cases reported eight recovered and seven died. "These rules were partly Dr. Willy Meyers's," he said "as indications for operation." (1) operate early, within twelve hours, in the acute perforative form; (2) take the pulse as a guide, 106 to 120 being the indication for operation; the pulse should also be out of proportion to the temperature; (3) in cases of doubt, operation is better than waiting; (4) sudden lull of symptoms, and within twelve hours, sudden recurrence; (5) in cases of old appendicitis lit up during pregnancy, operation ought to be done, even if the attack is a mild one, especially if it occurs early in pregnancy."

In the discussion, Dr. Robert T. Morris thought appendicitis should be expected more frequently during pregnancy, because, first, in such condition the appendix hung over the pelvic brim in about 35 per cent. of the cases and was thus more liable to become bruised by the enlarging uterus; and, second, in the many cases where adhesions existed, they were likely to be broken up during the same process. He thought that the catarrhal form could only exist as an extension of intestinal catarrh, and would give no symptoms of appendicitis. Resisting clonic spasm of the abdominal wall, he thought, served to distinguish acute appendicitis from typhoid and salpingitis.

Dr. William T. Lusk related a case where several experienced surgeons felt an appendix distinctly, which proved to be a tubal pregnancy upon operation.

Dr. H. J. Garrigues operated a case, which subsequently developed a fatal pleurisy. Dr. Willy Meyers thought that every recurrent case should be operated, to guard against hernia in the operation wound.

Dr. Howard Lilienthal related a case, wherein he was called in consultation by Dr. Vineberg, apparently of appendicitis complicating pregnancy, although the possibility of typhoid fever was not excluded. They found a normal appendix, and nothing to account for the symptoms. On emptying the uterus, the collapse became more profound, but she suddenly took a turn for the better.

Dr. Bernard S. Talmey thought that in perhaps not more than 10 per cent. of all cases, was it necessary to call in the surgeon; he thought the other 90 per cent. must be catarrhal.

Dr. Henry Illoway thought pressure of hard fecal masses accounted for the inflammation originating from within.

In the *American Medico-Surgical Bulletin* for November 7, 1896, the case of a 9-year-old boy was reported, where Dr. B. F. Curtis first treated a swelling of the foot with a wet dressing; a few days after an abscess of the biceps had to be incised, but the elevated temperature continued, and two days later an appendicitis developed, presenting upon operation a large encapsulated abscess, between the cecum and omentum; the general peritoneal cavity was necessarily opened. A septic bronchitis later developed, and it was decided that after recovery it would be advisable to operate for a ventral hernia.

Dr. Mundé, at the Congress of Physicians and Surgeons (*Medical Week*, May 28, 1897,) pointed out that abortion or premature labor was induced by the appendicitis, on account of local irritation, or through the fatal effect on the ovum of the hyperpyrexia; another point was the masking of the true diagnosis by normal labor-pains, and the difficulty of differentiating it from puerperal septic infection. He claimed that if labor was in progress, immediate operation was essential, in order to avoid the effect of traction-rupture of the abscess by the uterine contractions. He believed that vagino-abdominal palpation did not cause pain, unless salpingo-oöphorectomy coëxisted; and that a pyosalpinx or pusovary adherent to the peritoneum in the right iliac region, or a small ovarian cyst with twisted pedicle, might simulate it. If the diagnosis was in doubt he preferred making the incision in the right semilunar line, since, if it proved an appendicitis, this was the shortest way to the seat of disease; and, if salpingo-oöphoritis, pyosalpinx or ovarian abscess, the diseased organ could quite as easily be removed as through a median incision.

Pinard (*Sem. Med.*, March 23, 1898, per *British Medical Journal*, May 7, 1898,) has collected forty-five cases, the diagnosis being confirmed in 30 by operation or post-mortem. He concludes that the child dies as a rule very rapidly from infection—that it is only possible to save both mother and child when the abscess is limited and encysted; that every type of appendicitis may occur; that diagnosis is usually possible with care; that induction of labor is unjustifiable, since pregnancy is not always interrupted, if the mother recovers, and that relapsing cases should be operated previous to, or in the interval between, pregnancies.

We have now given a clear résumé of the literature on the subject, and have collected, besides, 1,024 contributions on appendicitis from our library, but lack of space prevents their enumeration here. We are convinced, also, that many cases have never been recorded, chiefly because, on the one hand, the course of the disease was too mild to excite interest, and on the other because a fatal result followed operation. The only way, there, fore, to arrive at an understanding of the best form of procedure to pursue in a given case, would be to address a written set of questions to every licensed practitioner, and to formulate a set of rules from the majority opinions, which all should be expected to adhere to, until revised.

The logic of conservatism in the treatment of appendicitis during the reproductive process, may be summed up as follows:

(1) The cardinal argument is the admitted fact that cases do recover completely without operation; (2) it is necessary to respect the popular fear of all operations, in the absence of good reasons for ignoring it; (3) "self-preservation is the first law of nature," and none of you desire another practitioner, with perhaps less conscientious scruples,—and perhaps as much in need of "the root of evil" as yourself,—to displace you by humoring the patient's feelings with a recommendation of delay, after you have insisted on immediate operation; (4) the still open question as to which procedure is safest for the mother, a child's life being the secondary consideration. And, finally, the knowledge that certain cases are beyond human assistance from the onset.

1547 MADISON AVE.

---

IN A discussion of the safest anesthetic to use in diseases of the heart, Hare (*American Journal of the Medical Sciences*, August, 1901,) states that the cardio-vascular system of patients who are going to be anesthetised should be more carefully studied than is customary. Ether greatly increases the arterial tension and where the latter is high the administration of ether is directly contraindicated.

